# Crosstalk between heterotrimeric G protein-coupled signaling pathways and WRKY transcription factors modulating plant responses to suboptimal micronutrient conditions

**DOI:** 10.1093/jxb/eraa108

**Published:** 2020-02-28

**Authors:** Ting-Ying Wu, Shalini Krishnamoorthi, Honzhen Goh, Richalynn Leong, Amy Catherine Sanson, Daisuke Urano

**Affiliations:** 1 Temasek Life Sciences Laboratory, Singapore; 2 Mathematical Sciences Institute, Australian National University, Canberra, Australia; 3 Department of Biological Sciences, National University of Singapore, Singapore; 4 Michigan State University, USA

**Keywords:** Arabidopsis, G protein signaling, meta-analysis, nutrient stress response, rice, transcription factors

## Abstract

Nutrient stresses induce foliar chlorosis and growth defects. Here we propose heterotrimeric G proteins as signaling mediators of various nutrient stresses, through meta-analyses of >20 transcriptomic data sets associated with nutrient stresses or G protein mutations. Systematic comparison of transcriptomic data yielded 104 genes regulated by G protein subunits under common nutrient stresses: 69 genes under Gβ subunit (AGB1) control and 35 genes under Gα subunit (GPA1) control. Quantitative real-time PCR experiments validate that several transcription factors and metal transporters changed in expression level under suboptimal iron, zinc, and/or copper concentrations, while being misregulated in the Arabidopsis Gβ-null (*agb1*) mutant. The *agb1* mutant had altered metal ion profiles and exhibited severe growth arrest under zinc stress, and aberrant root waving under iron and zinc stresses, while the Gα-null mutation attenuated leaf chlorosis under iron deficiency in both Arabidopsis and rice. Our transcriptional network analysis inferred computationally that WRKY-family transcription factors mediate the AGB1-dependent nutrient responses. As corroborating evidence of our inference, ectopic expression of *WRKY25* or *WRKY33* rescued the zinc stress phenotypes and the expression of zinc transporters in the *agb1-2* background. These results, together with Gene Ontology analyses, suggest two contrasting roles for G protein-coupled signaling pathways in micronutrient stress responses: one enhancing general stress tolerance and the other modulating ion homeostasis through WRKY transcriptional regulatory networks. In addition, tolerance to iron stress in the rice Gα mutant provides an inroad to improve nutrient stress tolerance of agricultural crops by manipulating G protein signaling.

## Teaser

Transcriptomic gene regulatory network analysis demonstrates two contrasting roles of the G proteins Gα and Gβ in micronutrient stress responses and ion homeostasis.

## Introduction

Insufficient or excessive levels of nutrients in the soil affect plant growth and development, eventually causing losses of crop yields. Plants cope with the suboptimal nutritional conditions with two major cellular responses, one is an element-specific response while the other is a general response independent of the types of elements. The element-specific response includes feedback regulation of divalent transporters and of uptake and/or translocation rates of specific ions, while the general response is more related to senescence and growth inhibition that occur at a later time point (Krämer, 2010; Kobayashi and Nishizawa, 2012). Arabidopsis transcriptome studies identified massive transcriptional changes under nutrient stresses, including membrane transporters, enzymes to synthesize metal chelators, and senescence-related genes (Brumbarova *et al.*, 2015). These transcriptional responses are mediated by nutrient-responsive transcription factors (TFs). For example, bZIP19 and bZIP23 regulate the expression levels of zinc transporters ZIP3 and ZIP4 under zinc deficiency treatment, leading to the modulation of shoot and root development and leaf chlorosis (Assunçao et al., 2010). While these comprehensive transcriptional networks have been deciphered under various nutrient stress conditions, little is known about how plant cells perceive nutritional changes and trigger the downstream transcriptional regulatory cascades.

Heterotrimeric G protein is composed of Gα, Gβ, and Gγ subunits, and regulates various physiological and developmental processes upon changes in phytohormone levels, environmental cues, and pathogen infection (Trusov and Botella, 2016; Urano *et al.*, 2016b). Upon extracellular stimuli, the G protein trimer separates into an active Gα subunit and Gβγ dimer, transmitting signals to downstream targets (Assmann, 2004; Urano *et al.*, 2013). Arabidopsis has two types of Gα subunits: canonical Gα (GPA1) and non-canonical extra-large Gα (XLG1, XLG2, and XLG3), both of which form a trimeric complex with the Gβγ dimer while being coupled with distinctive upstream regulators and downstream targets (Lee and Assmann, 1999; Ding *et al.*, 2008). Genetic studies demonstrated that loss-of-function mutations in G protein genes cause obvious developmental defects such as aberrant leaf and root elongation, meristem maintenance, and lateral root formation (Lease *et al.*, 2001; Ullah *et al.*, 2003; Chen *et al.*, 2006). Some of these developmental disorders in G protein mutants are similar to what was observed for wild-type (WT) plants under suboptimal nutritional conditions (Hindt and Guerinot, 2012; Giehl and von Wirén, 2014). In addition, null mutations in G protein subunit genes are known to alter sensitivity to a broad range of environmental stresses such as high salinity, drought, heavy metals, and cold temperature (Chakravorty *et al.*, 2015; Ma *et al.*, 2015; Urano *et al.*, 2016a). Given these previous findings with G protein mutants, we hypothesized that G protein signaling is an essential signaling mediator linking nutrient stresses to transcriptional regulation so as to determine shoot and root growth and morphology under suboptimal nutrient conditions in the soil.

In this study, we report signaling and transcriptional regulators: G protein complex and WRKY-family TFs that mediate plant nutrient responses. Comparative meta-analysis of transcriptomic data and quantitative PCR validation suggested that Arabidopsis Gβ had a higher association with phosphate, iron, zinc, and copper responses, while Gα signaling was independent from responses to suboptimal concentrations of the specific metal ions. Gα-regulated genes were more related to general abiotic stress while Gβ-regulated genes had molecular functions of cell wall biogenesis, transcriptional regulation, and phytohormone dynamics. These Gβ-regulated genes included WRKY TFs, well-known regulators of multiple abiotic and biotic stress responses. Metal profiles and physiological assays in Arabidopsis further found that Gβ-null mutants had a lower zinc content and exhibited drastic physiological and morphological changes under zinc deficiency or excess conditions. Moreover, Arabidopsis XLG- or Gβ-null mutants exhibited abnormal root waving under iron deficiency or excess zinc conditions, while the Gα-null mutation attenuated foliar chlorosis under iron deficiency in both Arabidopsis and rice. Our ectopic expression experiments further confirmed that *WRKY25* or *WRKY33* overexpression mitigates the zinc stress phenotypes, the feedback expression of zinc transporters, ZIP3 and ZIP4, in the Arabidopsis Gβ-null mutant. These lines of evidence suggested that physiological and transcriptional responses to nutrient deficiencies and excesses are mediated independently by two G protein subunits at least in part through WRKY-family TFs. In addition, the suppression of the canonical Gα pathway attenuates leaf senescence in crops as well as in Arabidopsis under nutrient stress conditions.

## Materials and methods

### Transcriptional meta-analysis: expression data source, normalization, pre-process, and analysis

**Fig. 1.**
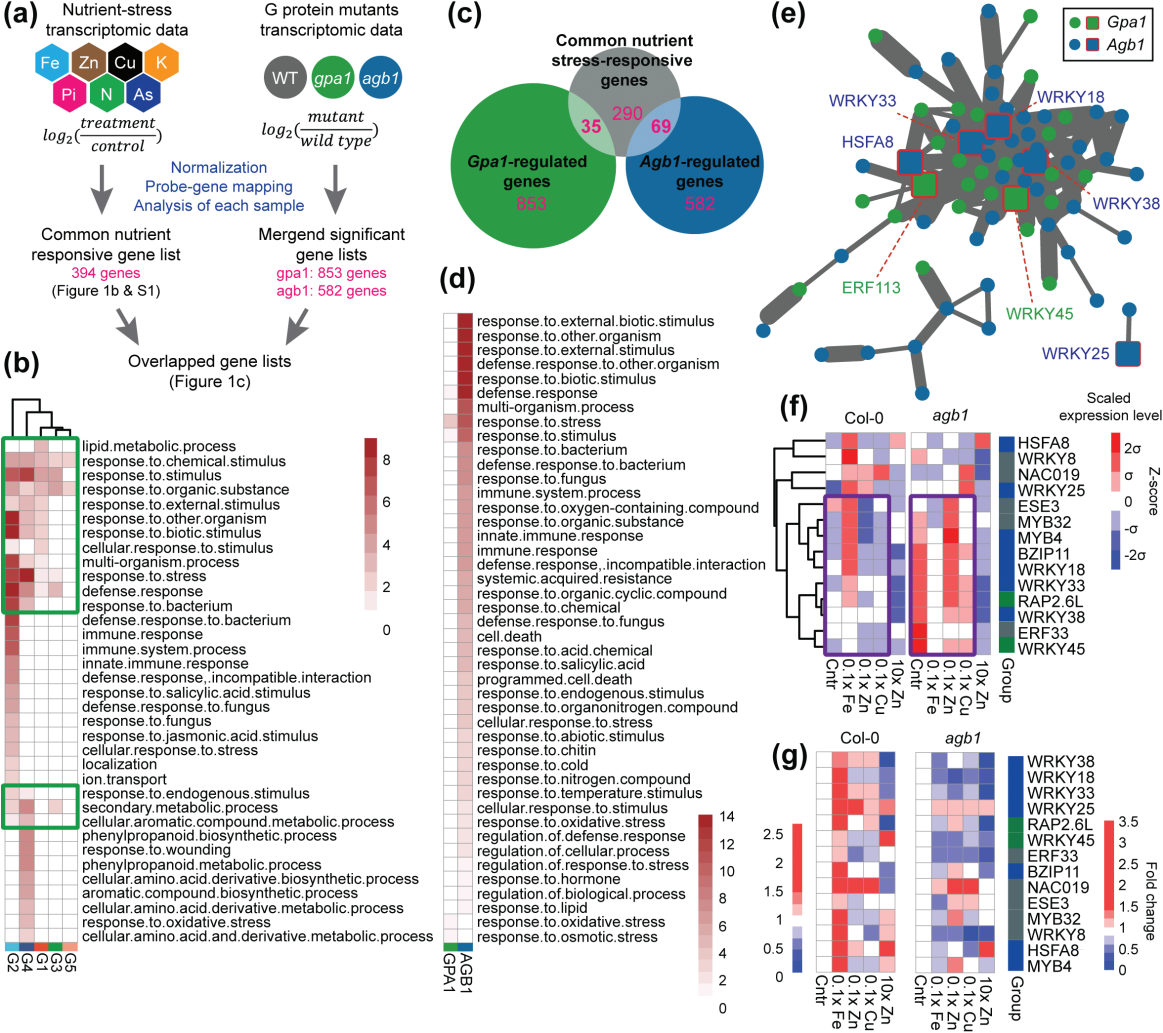
AGB1 regulates nutrient-responsive TFs under iron, zinc, and copper deprivation conditions. (a) Pipeline for transcriptomic meta-analysis and data normalization to identify transcriptional regulators under the control of G protein subunits and nutrient stresses. Nutrient-responsive genes and G protein-regulated genes were identified from public microarray ATH1 data sets in Arabidopsis. (b) GO analysis of the common nutrient-responsive genes. GO terms of biological process are shown. The color bar represents –log_10_(FDR) values. Five groups (G1–G5) were defined with a k-means clustering method as shown in Supplementary Fig. S2. Green boxes highlight GO terms that are enriched in two or more groups. (c) Venn diagram showing *gpa1*- and *agb1*-specific genes (green and blue circles) overlapping with common nutrient-responsive genes (gray circle) that are regulated by two or more types of nutrient stresses. (d) GO enrichment analysis of the *gpa1*- and *agb1*-specific genes. The color bar represents –log_10_(FDR). (e) Gene co-expression network of the overlapping *gpa1*- and *agb1*-specific genes constructed by WGCNA. TFs are presented in squares with a red outline, while other genes are in circles. Nodes representing *gpa1*- or *agb1*-specific genes are shown in blue or green, respectively. (f, g) qRT-PCR analyses of TFs selected from the commonly expressed gene list. Five-day-old seedlings of Col-0 and *agb1-2* were grown under iron, zinc, or copper deficiency, or zinc excess conditions, respectively. The heat map in (f) shows the mean of log_2_-transformed relative expression levels from three biological replicates. The data in (g) were normalized by individual gene expression under control condition from the WT and *agb1-2*, respectively, and are shown as fold change over each of the controls. Raw data, gene expression levels relative to *Act2*, are presented in bar graphs in Supplementary Fig. S6.

Data sets used in this study were obtained from the Gene Expression Omnibus (GEO) from the National Center for Biotechnology Information (NCBI) and Array-Express. The original design of the biological experiments selected and analyzed in this study meet the Minimum Information About a Microarray Experiment (MIAME) proposal (Brazma *et al.*, 2001). The GEO accession numbers and corresponding references are listed in Supplementary Table S1 at *JXB* online. The raw data were processed with quantile normalization and background correction using the robust multi-array average (RMA) by R packages ‘affy’ and ‘gcrma’ (Gautier *et al.*, 2004; Wu *et al.*, 2004). The ‘limma’ package (Smyth, 2005) was used to identify differentially expressed genes (DEGs) and compute log_2_-transformed values of the expression level in a linear model for subsequent network analysis. A gene was considered as a DEG if its log_2_-fold change (FC) value was >1 and significantly different at a *P*-value [false discovery rate (FDR)] of <0.05 from its corresponding control group. Affymetrix probe IDs were then converted to AGI locus identification numbers by the BAR website (Waese *et al.*, 2017). Probe IDs with no match or ambiguously matching multiple loci were discarded. In order to reduce the size effect and to obtain the moderate *P*-value, the DEGs from each data set were once more determined by the ‘MetaMA’ R package with default settings (Marot *et al.*, 2009). The common nutrient-responsive genes were then identified using a *P*-value <0.05 as cut-off. Principal component analysis (PCA) was applied to the common responsive DEGs to assess similarities and differences among different microarray data sets. Expression changes of the common nutrient-responsive genes were log_2_ transformed then clustered by the k-means method. In this meta-analysis, we compared *gpa1* and *agb1* mutants with WT Col-0 under control condition (Fig. 1a) with the aim of identifying genes that explain the distinct phenotypes of *gpa1* and *agb1* mutants under nutrient stresses. While the meta-analysis yielded 53 genes commonly regulated by both GPA1 and AGB1, these overlapping genes were not considered for further analysis. Note that Gene Ontology (GO) terms of the 53 genes were associated with oxidative stress, cell death, and defense response, but only had marginally significant adjusted *P*-values (0.04, 0.10, and 0.07, respectively).

The microarray data sets used for meta-analysis were generated under various nutrient stress conditions. The normalization method across data sets is described in Supplementary Fig. S1. Background correction and quantile normalization of Affymetrix CEL files were performed with the RMA expression measure using the Bioconductor ‘affy’ package (Gautier *et al.*, 2004). The log_2_FC value in expression was calculated for all data sets by pairwise comparison between treatment and control experiments for each nutrient stress and time point. Contrast matrices and linear model fits were created using R and the Bioconductor ‘LIMMA’ package (Ritchie *et al.*, 2015). The *P*-values for log_2_FC were corrected for multiple testing (adjusted *P*-values) using the BH (Benjamini–Hochberg) method (Benjamini and Hochberg, 1995).

### Gene clustering, and constructing and visualizing gene co-expression networks

The ‘WGCNA’ R package was used to construct gene co-expression networks from common nutrient-responsive genes. The node pairs in the co-expression networks were filtered by edge *P*-values calculated using Pearson’s correlation coefficient (adjusted *P*-value <0.0005) (Langfelder and Horvath, 2008). A TF-prioritized network was constructed using the GENIE3 and iRafNet R packages (Huynh-Thu *et al.*, 2010; Petralia *et al.*, 2015). The random forest method with 1000 decision trees and the information from conserved TF-binding sequence as well as known gene functions from the AraNet database were used for network construction (Lee *et al.*, 2015). The resulting network was then visualized using the Cytoscape software (Shannon *et al.*, 2003). The overlapping coefficient was calculated as |A∩ B|/min(|A|, |B|), where |A| and |B| represent the number of stress-related genes in stress A and B, respectively (Ma *et al.*, 2014). The overlapping coefficient ranges from 1 (high correlation) to 0 (no correlation).

### Gene Ontology analysis for biological function

GO analysis with a focus on biological process was performed using the AgriGO website (Tian *et al.*, 2017) with default settings. Significantly enriched GO terms are presented with a heatmap showing the –log_10_(FDR) values.

### Responses to iron and zinc deprivation and zinc excess in Arabidopsis

Arabidopsis seeds for WT Col-0 and G protein mutants were sterilized with 1.2% sodium hypochlorite for 10 min and then were stratified for 3 d at 4 °C. The seeds were germinated *in vitro* for 6 d in plates containing half-strength Murashige and Skoog (MS) medium (Murashige and Skoog, 1962) containing 0.8% purified agar at 22 °C horizontally. The 6-day-old seedlings of similar size were subsequently transplanted to plates with Yamagami medium with 0.8% purified agar (Li *et al.*, 2017). While the Yamagami medium for the control condition contains 8.6 µM Fe(III)-EDTA or 1 µM ZnSO_4_, no Fe(III)-EDTA or ZnSO_4_ was added into the Yamagami medium for the iron- or zinc-deficient condition. The plates were placed vertically at 22 °C with a 16/8 h light/dark cycle at 100 µmol photons m^−2^ s^−1^ and root tips were marked every 2 d after starting the treatments. The root elongation was then measured and quantified by ImageJ. The wavy root index was calculated by root length divided by the depth of the root (Vaughn and Masson, 2011).

Shoot phenotypes such as leaf size and foliar chlorosis were also tested using Yamagami media. One-tenth of the amount of Fe(III)-EDTA or ZnSO_4_ was added to the medium for iron or zinc deficiency conditions, while 10 times the amount of ZnSO_4_ was used for zinc excess conditions. For analysis of shoot phenotypes, sterilized seeds from Col-0 and mutant lines were germinated directly on the treatment plates, and the plates were kept horizontally for 12 d before being imaged. Green leaf area and chlorophyll index were analyzed with ImageJ as previously reported (Liang *et al.*, 2017). The numbers of plants showing sustained growth arrest or a black shoot apical meristem were counted manually, and divided by the total number of germinated plants.

### Responses to iron deprivation in rice

Rice NB and DK22 seeds were germinated for 3 d in Petri dishes with H_2_O and 10 μM gibberellic acid under darkness at 30 °C. Germinated seedlings were then transferred into containers holding 10 liters of de-ionized water for 7 d at 28 °C. The seedlings of similar height were then transferred to the containers with 10 liters of rice hydroponic solution. The nutrient solution was prepared as previously described (Kobayashi *et al.*, 2005). While the solution contains 100 µM Fe(III)-EDTA and 0.5 µM ZnSO_4_, 5 µM or 0.025 µM Fe(III)-EDTA or ZnSO_4_ was used for iron- and zinc-deficient conditions. The rice plants were treated for 14 d at 27–28 °C with 24 h continuous light. The pH of the nutrient solution was adjusted daily to 5.5 with 1 M HCl, and the nutrient solution was renewed weekly. Root lengths were measured by a ruler at day 10. Chlorophyll contents were measured by a CCM-300 chlorophyll content meter (Opti-Sciences) every 2.5 d from day 5 until day 10 after the treatment started. All statistical analyses were conducted in R (R Development Core Team, 2010).

### Measurement of ion contents in Col-0 and G protein mutants

Ion profiling was determined by following a previous publication with slight modification (Wu *et al.*, 2019). In brief, 18-day-old seedlings from Col-0 and G protein mutants from treatment plates (Yamagami medium with 1% purified agar) were dried at 60 °C for 3 d. A 25 mg aliquot of ground leaf samples was boiled in 10 ml of 65% (v/v) HNO_3_ solution at 120 °C for 90 min. A 3 ml volume of 30% (v/v) H_2_O_2_ was subsequently added and continuously boiled at 120 °C for 90 min. Metal concentrations were determined using inductively coupled plasma-optical emission spectroscopy (ICP-OES) (Varian Vista-MPX CCD Simultaneous ICP-OES) and expressed per gram of shoot dry weight. Data were analyzed using Student’s *t*-test. The criterion of *P*<0.05 was used to determine statistically significant differences between Col-0 and mutant lines.

### Plasmid construction and generation of transgenic plants

The full-length coding sequences (CDS) of *WRKY25* and *WRKY33* genes were amplified from Col-0 plants. Primers (5'–3') used for gene amplification were WRKY25_F_BP, ggggacaagtttgtacaaaaaagcaggcttcATGTCTTCCACTTCTTTCACCGA and WRKY25_R_BP, ggggaccactttgtacaagaaagctgggtcTCACGAGCGACGTAGCGC; WRKY33_F_BP, ggggacaagtttgtacaaaaaagcaggcttcATGGCTGCTTCTTTTCTTACA and WRKY33_R_BP, ggggaccactttgtacaagaaagctgggtcTCAGGGCATAAACGAATCGA. The CDS was then introduced into the binary vector containing 35S promoters that drive N-terminal red fluorescent protein (RFP)-tagged protein and a hygromycin selection marker. The flower dipping method was carried out as described in *agb1-2* plants (Zhang *et al.*, 2006). The positive overexpressing (OE) lines were selected on 1/2 MS medium containing 25 mg l^–1^ hygromycin. The gene expression of *WRKY25* and *WRKY33* was confirmed by quantitative real-time PCR (qRT-PCR), and all of the experiments were performed using the T_3_ generation.

### RNA extraction, cDNA synthesis, and qRT-PCR

Col-0, *gpa1-3*, and *agb1-2* seeds were germinated and grown for 5 d on Yamagami medium plates or treatment plates that contained a 10 times lower concentration of iron, zinc, or copper in the same medium. Total RNA was extracted from the 5-day-old seedlings as previously described (Wu *et al.*, 2013). The RNA was treated with RQ1 RNase-Free DNase (Promega, USA) to remove genomic DNA contamination. First-strand cDNA was synthesized using the GoScript™ Reverse Transcription Sytem kit (Promega, USA). qRT-PCR was performed as previously described (Wu *et al.*, 2013). In brief, qRT-PCR was performed in a CFX Connect™ Real-Time PCR Detection System (Bio-rad, USA) using the KAPA SYBR^®^ FAST qPCR Master Mix (2X) Universal kit following the manufacturer’s protocol (KAPA, USA). Each reaction was run in triplicate in a volume of 20 µl with an initial denaturation step at 95 °C for 10 min, followed by 40 cycles of 95 °C for 15 s and 60 °C for 60 s. Data were analyzed according to the manufacturer’s instructions using the Bio-rad Software v3.1 (Bio-rad, USA). The expression level of genes of interest was normalized to the expression of Arabidopsis *ACTIN 2* (*AtACT2*). All the primers used for qRT-PCR experiments are listed in Supplementary Table S4.

## Results

### Meta-transcriptome analysis revealed common responsive genes under nutrient stress

A large number of Arabidopsis transcriptomic data sets (see the Material and methods) enabled us to extract biological information that remained unaddressed by conventional genetic studies. To find a possible linkage between G protein signaling and nutrient responses in plant cells, we collected transcriptomic data sets obtained under various nutrient conditions and compared them with transcriptomic changes observed in Arabidopsis *gpa1* or *agb1* mutants. The bioinformatics pipeline for meta-transcriptome analysis is illustrated in Fig. 1a and Supplementary Fig. S1. After a search in databases and quality control analysis, normalized microarray data were selected from 23 independent experiments that analyzed nutrient stresses in different categories, namely iron (Fe), zinc (Zn), potassium (K), and phosphate (Pi) deficiency as well as cadmium (Cd), arsenate (As), and zinc excess (Fig. S1; Supplementary Table S1). Iron, phosphate, and zinc deficiency, and zinc excess treatments had the greatest numbers of DEGs, namely 4078, 3504, 1306, and 2476 DEGs, respectively (Fig. S1a; Supplementary Table S1). Among >5000 unique genes identified from the microarray data sets, 394 genes changed in expression level commonly under two or more types of nutrient stresses (Supplementary Fig. S2). The similarity and differences of global transcriptomic changes caused by different metal stresses were further analyzed using PCA (Supplementary Fig. S2a) and overlapping coefficient (Supplementary Fig. S3b). These analyses suggested that iron, copper, or phosphate deficiency were grouped together, while zinc excess was clustered with cadmium and arsenate excess. Similar results were yielded by overlapping coefficient analysis, where iron, copper, and phosphate show high overlapping coefficients (Supplementary Fig. S3b).

We next applied k-means clustering analysis to the 394 genes and classified them into five groups (Fig. 1b). GO analysis found that the common nutrient-responsive genes have molecular functions related to cell wall organization, abiotic and biotic stress response, immune response, cell death, hormone response, and transportation (Fig. 1b). These GO terms largely overlapped with AGB1-regulated signaling pathways under stress conditions (Pandey *et al.*, 2010; Delgado-Cerezo *et al.*, 2012), hence a comparative meta-analysis was conducted to evaluate commonalities between G protein-regulated and nutrient-responsive genes. Differential gene expression caused by Gα-null (*gpa1-3*) and Gβ-null (*agb1-2*) mutations were isolated from public microarray data (Supplementary Figs S1–S3; Supplementary Table S1), yielding 888 *gpa1*-specific or 651 *agb1*-specific genes. A Venn diagram shows the overlap of common nutrient stress genes and G protein-regulated genes (Fig. 1c; Supplementary Tables S2, S3), where common nutrient stresses share 35 genes with a *gpa1-3* mutation and 69 genes with an *agb1-2* mutation [hypergeometric *P*-value=0.078 (*gpa1-3*) or 1.62×10^−19^ (*agb1-2*), respectively]. The GO terms of *gpa1*- and *agb1*-specifc genes indicate a limited overlap between Gα- and Gβ-dependent functions under nutritional stress conditions (Fig. 1d). Specifically, AGB1-regulated genes are related to biotic and abiotic stress response, secondary metabolism, and programmed cell death, whereas GPA1-regulated genes are related more to abiotic stress response, including oxidative and osmotic stress response.

### TFs commonly regulated by G proteins and nutrients

In gene regulatory networks, a few influential genes such as those encoding TFs regulate expression patterns of most other genes. Those highly influential genes are connected to many other genes in co-expression networks, hence they are represented as hubs. To visualize such influential factors from the 394 common nutrient-responsive genes, a co-expression network and a TF-prioritized semi-supervised network were constructed with weighted correlation network analysis (WGCNA) and GEne Network Inference with Ensemble of trees (GENIE3) methods (Langfelder and Horvath, 2008; Huynh-Thu *et al.*, 2010). The networks contained several hub TFs including WRKY, MYB, NAC, HSF, and bHLH (Supplementary Fig. S2c, d). Similarly, another co-expression network was built using 104 genes that are misregulated by both G protein mutations and nutrient stresses (35 *gpa1*-specific and 69 *agb1*-specific genes shown in Fig. 1e), and we found that two types of TFs, HSFA8 and WRKYs, were connected to most other genes in the network (Fig. 1e), suggesting that these TFs are induced by common nutrient stresses under the control of trimeric G protein. To validate the findings, we further analyzed the expression of 14 TF genes using qRT-PCR under control, iron, copper, and zinc deficiency, as well as zinc excess conditions (Fig. 1f). In control medium, most of the TF genes were expressed at higher levels in *agb1-2* plants than in WT plants, but their expression was significantly different under nutrient stress conditions (Fig. 1f). All the TF genes were differentially expressed by various stress conditions in WT plants, >2-fold higher or lower compared with the control condition (Fig. 1g). However, the expression pattern of the selected TF genes was opposite in *agb1-2* under stress conditions (Fig. 1g). The expression of WRKY18, 33, and 38 showed the most striking differences between the WT and *agb1-2* (Fig. 1g). Interestingly, these genes were identified as hub genes in the co-expression network constructed from the nutrient and G protein transcriptomic meta-analysis (Fig. 1e). The TFs misregulated in *agb1-2* had molecular functions related to stress tolerance, hormone regulation, and response to reactive oxygen species (ROS). For example, WRKY18 and 38 are known as critical players in the defense response, and WRKY25, WRKY33, and HSFA8 are involved in regulation of ROS production (Giesguth *et al.*, 2015; Phukan *et al.*, 2016; Yabuta, 2016). NAC and MYB TF families play important roles in various nutrient stresses (Nuruzzaman *et al.*, 2013; Briat *et al.*, 2015), and ETHYLENE-RESPONSIVE TRANSCRIPTION FACTOR 113 (ERF113, RAP2.6L) is a positive regulator of stress-induced senescence (Liu *et al.*, 2012). The qRT-PCR results were consistent with the transcriptomic meta-analysis (Fig. 1), and suggested that WRKY and other TFs were transcriptionally regulated by nutrient conditions downstream of AGB1.

### Metal homeostasis in Arabidopsis *gpa1* and *agb1* mutants

**Fig. 2.**
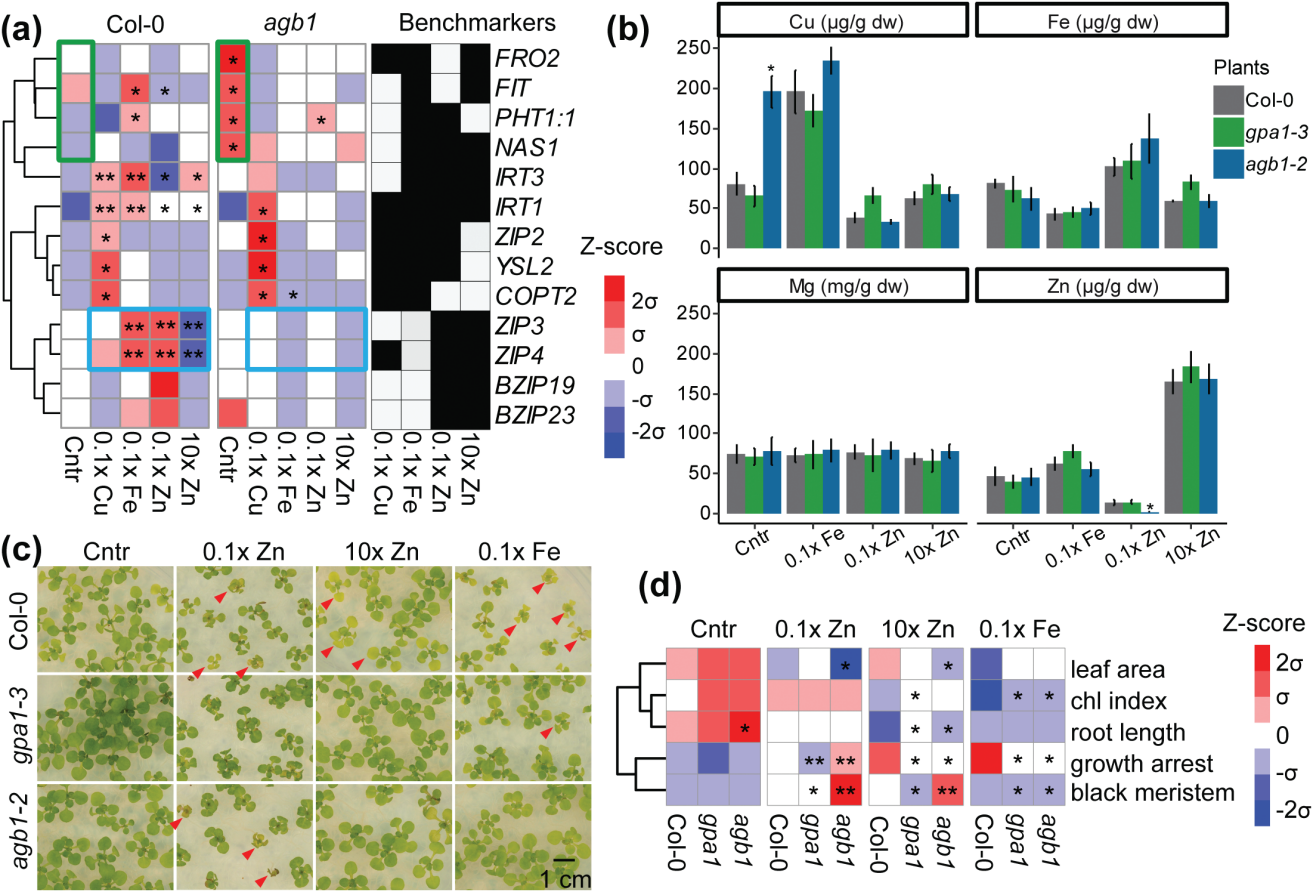
*agb1* mutation alters adaptive responses to iron deprivation, zinc deprivation, and zinc excess conditions. (a) qRT-PCR analyses of marker genes. Roughly 100 Col-0 and *agb1-2* 5-day-old seedlings were pooled for RNA extraction from iron, zinc, and copper deficiency, and zinc excess conditions. The heat map shows the mean of log_2_-transformed relative expression levels from three biological replicates. The black filled squares indicate that marker genes exhibited differential expression under certain stress conditions. The blue box highlights zinc markers that are misregulated significantly in *agb1-2*. The green box shows iron-responsive genes up-regulated in *agb1-2* under the control condition. Asterisks indicate significant differences from Col-0 with the control treatment. *P<0.05 and **P<0.01. (b) Metal contents in WT and *agb1-2* plants grown in control, iron and zinc deficiency, and zinc excess conditions. The metal contents (mg or μg g DW^–1^ of tissue) were determined by ICP-OES from 18-day-old seedlings. Data shown are the mean of two different biological replicates with the SD. Student’s *t*-test was used to determine significant differences. *P<0.05. (c) Representative photos of phenotypes shown in Col-0, *gpa1-3*, and *agb1-2* plants. Photos were taken after 12 d of treatment. Red arrowheads indicate plants that exhibit growth arrest or chlorotic leaves. (d) Quantitative data analyzed from phenotypic assay. The heatmap indicated a decrease (blue) or an increase (red) with respect to the mean value of three genotypes (Col-0, *gpa1-3*, and *agb1-2*) under control, iron and zinc deficiency, and zinc excess conditions. Each row represented individual physiological traits ordered by the hierarchical clustering algorithm. Chl denotes chlorophyll. Original physiological data are available in Supplementary Table S5. Asterisks in (b) and (d) represent significant differences from the WT group for each stress treatment.

Our meta-analysis revealed that *agb1*-specific genes had a higher overlapping coefficient with iron, copper, and zinc deficiency as well as the zinc excess condition (Supplementary Fig. S1), indicating that G proteins may play roles in metal homeostasis *in planta*. We further analyzed the expression of marker genes including ion transporters and TFs for metal stresses in WT and *agb1-2* plants. Some of the marker genes were responsive to various divalent cations, while others were more specific to iron or zinc concentration in the WT (Fig. 2a). The expression pattern of *IRT1*, *IRT3*, *ZIP2*, *YSL2*, and *COPT2* was comparable in *agb1-2* and WT plants when exposed to various metal stresses (Fig. 2a). Interestingly we observed that the iron-responsive genes *FRO2*, *FIT*, *PHT1:1*, and *NAS1* were up-regulated significantly in the *agb1-2* mutant without stress (framed by a green box, Fig. 2a). On the other hand, zinc transporter genes *ZIP3* and *ZIP4* showed lower induction under zinc-deficient conditions in *agb1-2* (framed by a blue box, Fig. 2a). The misregulated expression of these ion transporters could lead to poor nutrient uptake and nutrient depletion in the shoot tissues. In addition, determination of metal concentrations in the shoots of WT, *gpa1-3*, and *agb1-2* plants revealed that the ion content in *gpa1-3* was similar to that in WT plants when exposed to different concentrations of metals whereas the zinc concentration in the shoot of *agb1-2* plants was >6-fold lower than in WT or *gpa1-3* plants under zinc-deficient condition (Fig. 2b).

### Shoot and root phenotypes of Arabidopsis *gpa1* and *agb1* mutants under iron and zinc deprivation and zinc excess conditions

Metal profiles and expression patterns of marker genes suggested that AGB1 controls plant responses to specific metals including iron and zinc, but not common responses to divalent cations. To investigate the crosstalk between G protein signaling and nutrients at the physiological level, we measured foliar chlorosis and developmental defects under iron and zinc deprivation and zinc excess conditions (Fig. 2c, d). *agb1-2* mutants had 27% or 19% smaller leaf area than WT plants under zinc-deficient or zinc excess conditions (Supplementary Table S2). Similarly, *agb1-2* plants exhibited growth arrest and damaged meristems under zinc-deficient conditions more severe than WT and *gpa1-3* plants (Fig. 2c, d). On the other hand, *gpa1-3* showed a higher chlorophyll index and circumvented growth inhibition as compared with the WT under various metal stresses (Fig. 2d; Supplementary Table S5), indicating that *gpa1-3* mutation improves plant tolerance to general nutrient stresses.

**Fig. 3.**
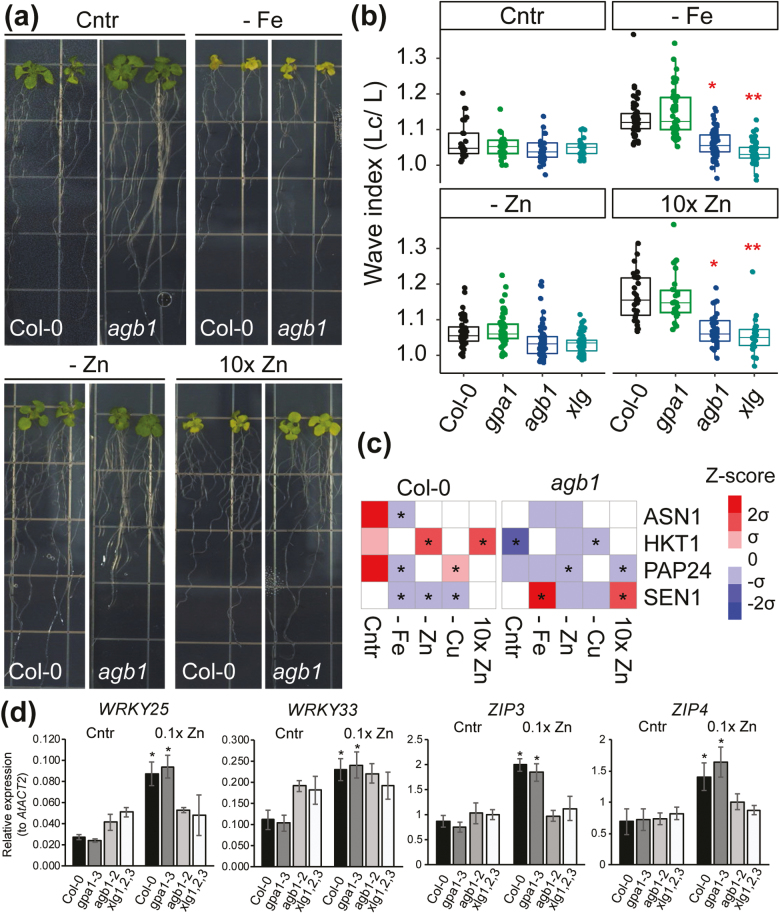
*AGB1* and *XLG* mediate root waving under iron-deficient and zinc excess conditions. (a) Representative images of Col-0 and *agb1-2* plants grown for 12 d vertically on Yamagami medium under control, iron and zinc deficient, and zinc excess conditions. (b) Wavy root index of Col-0, and *gpa1-3*, *agb1-2*, and *xlg1,2,3* mutants in Arabidopsis under control, iron deprivation, zinc deprivation, and zinc excess conditions. Raw data and a box plot are displayed for each genotype. One-way ANOVA followed by Tukey’s multiple comparison test was used to determine significant differences. *n*=30–40 for each genotype under each treatment *P<0.05, **P<0.01. The median and the 10th, 25th, 75th, and 90th percentiles are plotted. (c) qRT-PCR analyses of root wave-associated genes in Col-0 and *agb1-2* seedlings. Color codes in the heat map represent relative expression levels as *z*-scores. Rows or columns represent individual marker genes or nutrient treatments used in this analysis. Note that original root images on plates and root length data are available in Supplementary Fig. S4. Asterisks in (c) represent significant differences from the control group in Col-0. *P<0.05, **P<0.01. (d) Expression levels of *WRKY25*, *WRKY33*, *ZIP3*, and *ZIP4* in Col-0, and *gpa1-3*, *agb1-2*, and *xlg1,2,3* mutants. Five-day-old seedlings were grown on control (Cntr) or zinc deficiency (0.1× Zn) Yamagami medium. *AtACT2* was used as a reference gene. *P<0.05. *n*=3.

In addition to growth arrest and foliar chlorosis, nutrient availability and environmental disturbance affect root morphology such as root length, waving, and skewing. We tested the effect of iron deficiency, zinc deficiency, and zinc excess on the growth and waving of primary roots in WT and *gpa1*, *agb1*, and *xlg1,2,3* mutant plants (Fig. 3a–c; Supplementary Fig. S4). All tested treatments inhibited primary root growth in WT and mutant plants although to different extents (Fig. 3a; Supplementary Fig. S4). While the primary root length of the *agb1-2* mutant was 17% longer than that of Col-0 on our control medium, the length was comparable with that of other genotypes under zinc or iron deprivation conditions (Supplementary Fig. S4a). Root waving was observed only on iron deficiency or zinc excess medium (Fig. 3b). Under these conditions, the wavy root index (primary root length divided by root depth) was 1.2-fold higher in the WT and *gpa1-3* than that in *agb1-2* and *xlg1,2,3*, suggesting that AGB1 is coupled with XLG rather than with GPA1 in these nutrient stress responses. Gene expression analysis further supported this coupling specificity (Fig. 3d). While WT and *gpa1-3* plants showed the feedback expression of two *WRKY* genes and two zinc transporters under zinc deficiency conditions, *agb1-2* and *xlg1,2,3* mutants completely failed to induce those genes.

We next analyzed the expression levels of marker genes for root waving in WT and *agb1-2* plants grown with various micronutrient stresses (Fig. 3c). The expression levels of *ASN1*, *HKT1*, *PAP24*, and *SEN1* were up- or down-regulated under metal stresses in the WT, which was consistent with previous studies; however, the expression patterns of these marker genes were altered in *agb1-2* (Fig. 3b) (Schultz *et al.*, 2017). In summary, expression levels of the wavy root-responsive genes were altered by nutrient availability and influenced by *agb1-2* mutation, suggesting thqat AGB1 has a major role in these transcriptional regulations linking extracellular metal concentrations to plastic root developments.

### Overexpression of *WRKY25* and *WRKY33* rescued zinc deficiency and excess phenotypes in *agb1-2* mutant

**Fig. 4.**
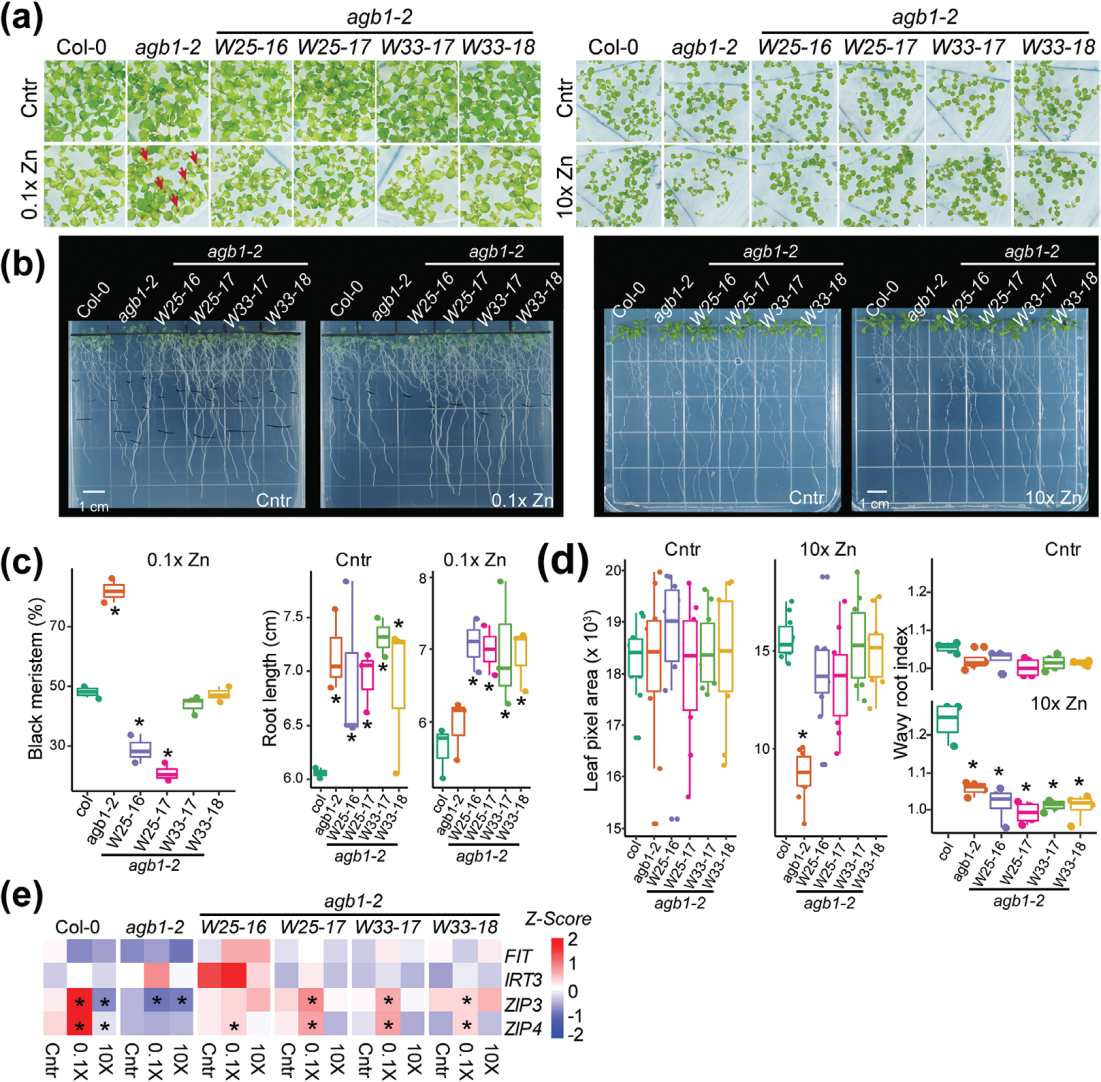
WRKY overexpression rescues Zn stress phenotypes of the *agb1-2* mutant. (a) Representative images of Col-0, *agb1-2*, and WRKY OE rescue lines (W25-16, W25-17, W33-17, and W33-18 in the *agb1-2* background). The seeds were directly grown on zinc deficiency (0.1× Zn) or zinc excess (10× Zn) medium for 12 d and 5 d, respectively. The photos were taken at 5 d (10× Zn) and 12 d (0.1× Zn). (b) Representative root images of Col-0, *agb1-2*, and WRKY rescue lines after 7 d treatment under zinc deficiency or excess conditions. The seeds were grown on 1/2 MS for 5 d and subsequently transferred to the treatment plates. (c, d) Quantitative data analyzed from physiological assays under zinc deficiency or excess conditions. The median and the 10th, 25,th 75th, and 90th percentiles are plotted. Asterisks denote significant differences compared with Col-0 at the *P*-value of *P<0.05; **P<0.01. The method of physiological analysis is mentioned in Fig. 2 and the Materials and methods. (e) qRT-PCR analyses of iron and zinc transporter genes. A heatmap shows relative expression levels of *FIT*, *IRT3*, *ZIP3*, and *ZIP4* genes as *z*-scores. Asterisks represent significant differences from expression of that gene in Col-0 plants under control conditions at a *P*-value of 0.05.

Our meta-transcriptome analyses, combined with experimental verification with qRT-PCR, inferred WRKYs as key TFs that would mediate zinc deficiency pathways downstream of AGB1. These WRKY TFs were highly induced by iron or zinc deficiency in WT Col-0, but not in the *agb1-2* mutant (Fig. 1g). To investigate the genetic interaction of these *WRKY* genes with *AGB1* in metal stress responses, we ectopically expressed *WRKY25* and *WRKY33* in the *agb1-2* mutant background and examined zinc deficiency phenotypes. Two independent lines of *WRKY25* and *WRKY33* were selected for further analysis based on their expression level (Supplementary Fig. S5). While >80% of *agb1-2* seedlings exhibited browning of the meristem under zinc-deficient conditions, the *WRKY25* OE lines (*W25-16* and *W25-17*) and *WRKY33* OE lines (*W33-17* and *W33-18*) showed only 20–30% and 50% of seedlings with a damaged meristem after zinc deficiency treatment (Fig. 4a, c). Overexpression of *WRKY* genes also rescued the reduced plant size phenotype in *agb1-2* under zinc excess conditions (Fig. 4a, d).

We next analyzed root morphological phenotypes of the *WRKY* OE lines. The *agb1-2* mutant, *WRKY25* OE, and *WRKY33* OE lines similarly exhibited 1.3-fold longer primary roots than the WT under control conditions (Fig. 4c). The WRKY OE lines maintained their long root phenotype even under zinc deficiency treatment, in contrast to *agb1-2* decreasing the root length by 14% (Fig. 4b, c). These results suggest that WRKY-family TFs only mediate the zinc stress response downstream of AGB1 but not the AGB1-dependent root development. However, the *WRKY* rescue lines formed significantly fewer root waves than Col-0 plants did under zinc excess treatment, rather similar to the root waves of *agb1-2* plants (Fig. 4b, d). Furthermore, qRT-PCR analysis demonstrated that these WRKY OE lines had higher expression of *ZIP3* and *ZIP4* transporters compared with the *agb1-2* mutant especially under zinc-deficient conditions (Fig. 4e). This suggested that *WRKY25* and *WRKY33* act downstream of Gβ and are partially involved in the regulation of *ZIP3* and *ZIP4* transcription.

### Gα-null mutation confers tolerance to iron deficiency in rice

**Fig. 5.**
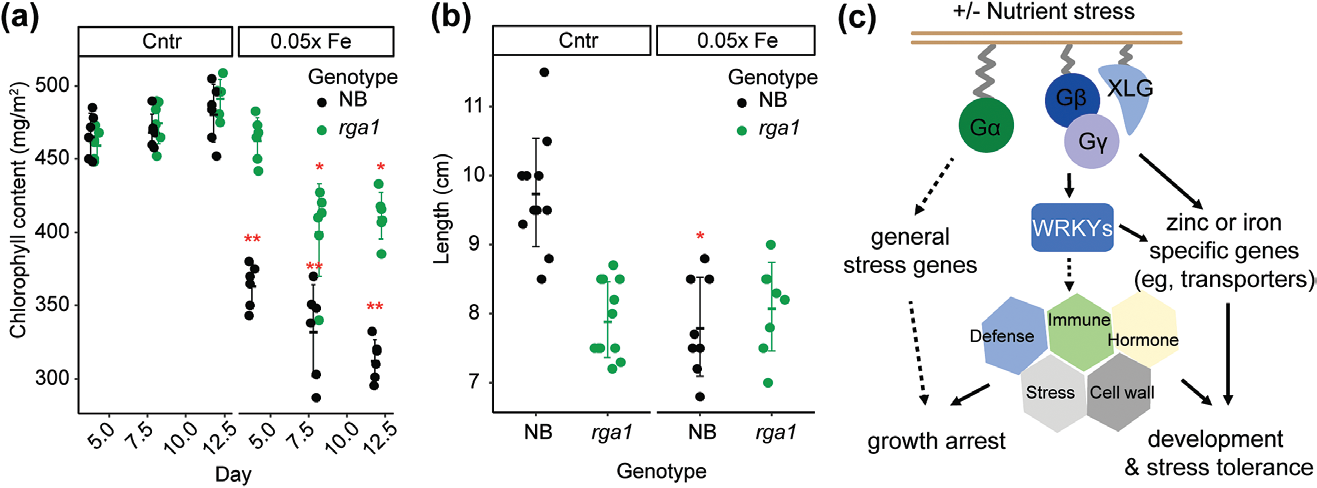
Gα mutation confers tolerance to iron deficiency in rice. (a) Chlorophyll content and (b) root lengths of NB and DK22 (*rga1*) under control or iron-deficient conditions. Raw data, the mean, and the SD are shown. One-way ANOVA was used to calculate the *P*-value. *n*=6 for each genotype under each treatment in (a) and 8–12 in (b). *P<0.05, **P<0.01. The error bars show SDs, and the middle lines are the mean value. *P*-value was determined between the control condition (Cntr) and the treatment condition (–Fe) on the same treatment day in (a), and between Cntr and the –Fe condition among the same genotype in (b). (c) A working model. When plants are exposed to suboptimal nutrient conditions, Gβγ dimer induces or suppresses the expression of downstream TF genes (*WRKY* genes) and common stress-responsive genes. Genes downstream of Gβ are closely associated with cell wall organization, immune response, and stress hormones, leading to developmental changes and stress adaptations depending on the types of metal ion stresses. In contrast, Gα regulates the expression of general stress response genes related to enhanced cell death and growth arrest. Genes downstream of Gα are barely affected by nutrient stresses, suggesting that those genes control a wide range of stress adaptation processes rather than nutritional stress-specific changes.

In contrast to the increased sensitivity to nutrient stresses in *agb1-2*, the *gpa1-3* mutant exhibited tolerance to nutrient stresses that is favorable for agricultural applications (Fig. 2). However monocot and eudicot plants are known to have, at least in part, different mechanisms to cope with nutrient stresses (Kobayashi and Nishizawa, 2012). To investigate if the Gα-null mutation results in any different phenotypes in staple crops, phenotypic data from the rice *rga1* mutant, DK22, and its WT (NB) plants were obtained under iron-deficient conditions. In iron-deficient conditions, the NB plants started showing chlorosis after 5 d of treatment whereas DK22 showed no chlorotic symptoms until day 8. Similarly, the chlorophyll content in NB was much lower as compared with that in DK22 throughout the iron treatment (Fig. 5a). In normal conditions, the root length of NB was 30% longer as compared with that of DK22. However, there is no significant difference in root length between NB and DK22 under iron-deficient conditions (Fig. 5b). This suggested that the Gα-mediated stress tolerance is conserved in these two species.

## Discussion

Through meta-analyses of transcriptome data, we show that a trimeric G protein, more specifically the AGB1 subunit, is involved in decoding extracellular nutritional conditions and controls ion homeostasis, shoot growth, foliar chlorosis, and root waving. These phenotypic alterations exhibit excellent correlation to transcriptional changes of metal homeostasis genes, marker genes for cell wall organization, and stress-related TFs. A working model in Fig. 5c summarizes possible G protein to WRKY pathways in response to nutritional changes. In this model, Gβγ, possibly together with the non-canonical XLGα, senses nutritional status, controls the expression levels of stress-responsive WRKY TFs, and alters ion homeostasis and other biological functions (Fig. 5c). Contrarily, the canonical Gα pathway regulates cell senescence activity in plants independent of nutrient conditions.

Our transcriptomic analyses revealed that nutrient stresses and the *agb1* mutation share a large number of common DEGs (Fig. 1c) including WRKYs and other TFs (Fig. 1e). While expression of these TF genes was greatly induced or suppressed under various metal stresses in WT plants (Fig. 1f, g), their basal expression and fold induction/suppression levels were drastically altered in *agb1-2* (purple boxes in Fig. 1f). Besides the drastic expression changes of TFs in *agb1* mutants, the marker genes for zinc homeostasis were specifically down-regulated in the *agb1-2* mutant (Fig. 2a). Among the gene markers, *ZIP3* and *ZIP4* encode zinc transporters regulated by two TFs, BZIP19 and BZIP23 (Fig. 2a). A previous study showed that zinc homeostasis is disturbed in the *bzip19/23* loss-of-function mutants, resulting in the misregulated expression of *ZIP3* and *ZIP4*, shorter root length, smaller rosettes, and cell death (Assunçao et al., 2010). In the *agb1*-2 mutant, the zinc concentration was lower than that in the WT and *gpa1-3* under zinc-deficient conditions (Fig. 2b), and the *agb1-2* mutation also caused poor growth including smaller leaves and shorter root length (Fig. 2c, d), resembling *bzip19* or *bzip23* mutant phenotypes. Hence, we argue that the zinc phenotypes observed in *agb1-2* would be associated with the imbalance of zinc homeostasis under zinc deficiency. Moreover, excessive zinc or cadmium concentration causes iron and phosphate deficiency phenotypes such as chlorosis and reduced biomass. Consistent with earlier reports, our meta-analysis indicated that DEGs from excess zinc or cadmium treatments were largely overlapping with DEGs from iron deficiency and showed a higher coefficient of overlapping (Supplementary Fig. S3). The excellent overlap of DEGs (Supplementary Fig. S3a), along with similar physiological responses to zinc excess and iron deficiency in the *agb1-2* mutant (Figs 2, 3), suggests that AGB1 could mediate nutritional crosstalk between deprivation or excess of these nutrients. While this work mainly focuses on zinc and iron as a model case, G protein signaling is associated with physiological responses to other nutrients such as carbon, phosphate, and nitrogen, and toxic heavy metals (Chakraborty *et al.*, 2015; Urano *et al.*, 2016a; Liang *et al.*, 2017). It will be interesting to pursue whether a similar molecular mechanism mediates other nutrient responses.

Our genetic complementation experiments confirmed that WRKY-family TFs mediate this AGB1-dependent nutrient response including foliar chlorosis, root elongation, and expression of metal transporters. Interestingly, the ectopic expression of WRKY25 or WRKY33 did not affect the basal expression levels of *ZIP3* and *ZIP4* transporters, while it restored the capability of Arabidopsis plants to induce or repress expression of these transporters in response to zinc deficiency and excess treatments (Fig. 4). Similarly, the WRKY25 or WRKY33 OE lines rescued the nutrient-dependent physiological responses of *agb1-2* such as plant size and cell death, but not the root morphology of *agb1-2* which is independent of nutrient stresses (longer primary root and fewer root wave phenotypes under normal conditions, Fig. 4c, d). The G protein complex is a well-known regulator of cell proliferation and plant morphological changes, in addition to modulating stress responses (Urano *et al.*, 2016b). Based on our experimental results, we propose that WRKY-family TFs are involved selectively in nutrient stress signaling rather than mediating both developmental and stress response pathways downstream of the G protein complex. To understand how G protein signaling and WRKYs coordinately function under nutrient stresses, future research may discover the target genes of WRKY25 and WRKY33 under different types of nutrient stresses. A previous study reported that WRKY33 binds directly to and regulates several ion transporter genes such as those encoding nitrate transporters, *WR3* and *NRT2.1*, a cation transporter, *CAX4*, a potassium transporter, *KT1*, and a zinc transporter, *ZIP5*, under a non-stress condition (Liu *et al.*, 2015; Birkenbihl *et al.*, 2017). Interestingly, the target genes of WRKY33 included *AGB1* and several *WRKY* genes, which may suggest a feedback regulation within WRKYs and between WRKYs and AGB1 (Liu *et al.*, 2015; Birkenbihl *et al.*, 2017). Because both G protein complex and WRKY-family TFs control multiple abiotic and biotic stresses in addition to nutrient homeostasis (Briat *et al.*, 2015; Liu *et al.*, 2015; Mudgil *et al.*, 2016; Birkenbihl *et al.*, 2017; Escudero *et al.*, 2017), a similar genetic interaction between G protein and WRKY-family TFs might exist in other stress pathways.

We propose a working model in which hormone changes complete a missing link between *agb1* mutation, nutrient stress, and root waving (Fig. 3a, b). It is known that bending of the root tips involves asymmetrical growth of the cell wall, achieved by asymmetric distributions of hormones (Roy and Bassham, 2014). The *agb1-2* plants failed to produce root waves when grown on inclined iron deficiency or zinc excess plates (Figs 3c; Supplementary Fig. S4), and changed the expression of four maker genes associated with wavy roots (Fig. 3c). These phenotypes resemble the effects of ethylene and auxin on root waving (Pandey *et al.*, 2008). In addition, various hormones including auxin, gibberellic acid, and cytokinin are known to be associated with Gβ-mediated root development in Arabidopsis and rice (Ullah *et al.*, 2003; Gao *et al.*, 2019). Ethylene accumulates significantly under iron, zinc, and other nutrient deficiencies and excesses (García *et al.*, 2015; Lucena *et al.*, 2015; Thao *et al.*, 2015). Hence, the reduced root waving in *agb1-2* under iron deficiency and zinc excess conditions could be due to the impairments in ethylene production or perception. Future studies might focus on examining the plausible interactions between trimeric G protein and phytohormone signals under nutrient stresses.

In addition to AGB1-regulated genes, transcriptional meta-analysis identified multiple senescence-related genes enriched in the *gpa1-3* mutant (Fig. 1d); however, in contrast to *agb1-2*, the majority of the GPA1-regulated genes were not induced or suppressed by nutrient stresses (Fig. 1c). This suggests that nutrient stress tolerance associated with Gα mutation is relevant to signaling networks or pathways modulated for general stress responses independently of nutrient status. Previous studies showed that Gα mutation in rice, maize, and Arabidopsis decreased chlorotic symptoms induced by various biotic and abiotic stresses, although the phenotype in the Arabidopsis *gpa1* mutant was observed only to a very low extent (Urano *et al.*, 2013). Our rice experiments showed that loss-of-function mutation in a Gα gene (DK22) greatly reduced foliar chlorosis under iron deficiency (Fig. 5a). The delayed chlorosis and higher chlorophyll content in the rice *rga1* mutant (DK22) as well as the Arabidopsis *gpa1* mutant may well be considered as part of general stress responses. The transcriptomic profiles of GPA1-regulated genes in Arabidopsis support these phenotypic outcomes, as they overlap less with nutrient-responsive genes but are highly associated with GO terms of abiotic stress responses.

Taken all together, our work suggests that physiological responses to nutrient deficiencies and excesses are mediated by Gα and Gβ subunits of heterotrimeric G protein, but the two subunits exert two opposing regulatory functions in the plant nutrient response. A considerable amount of agricultural soil contains suboptimal amount of iron, zinc, and other nutritional elements due to both natural processes and anthropological activities, hence the genetic manipulation of these G protein pathways may be a suitable strategy for the development of nutrient stress-tolerant crops. Our experimental results with Gα-null rice serve as solid evidence for such a strategy.

## Supplementary data

Supplementary data are available at *JXB* online.

Fig. S1. Pipeline of data normalization and analysis in this study.

Fig. S2. Transcriptional regulators for different types of nutrient stresses.

Fig. S3. Degree of overlap between *gpa1*-specific and *agb1*-specific genes with each nutrient-specific gene.

Fig. S4. Root length of Col-0 and G protein mutants under iron deficiency, zinc deficiency, and zinc excess in Arabidopsis.

Fig. S5. Expression of *WRKY25* and *WRKY33* of WRKY OE lines in the *agb1-2* background.

Fig. S6. qRT-PCR analyses of TFs selected from a list of commonly expressed genes.

Table S1. Sources and experimental conditions of microarray data used for this study.

Table S2. List of common nutrient-responsive genes.

Table S3. List of *gpa1*- and *agb1*-specific genes.

Table S4. List of primers used for qRT-PCR experiments.

Table S5. Shoot and root phenotypes under low or excess iron and zinc conditions.

eraa108_suppl_supplementary_figures_S1-S6_tables_S1_S4-S5

eraa108_suppl_supplementary_tables_S2-S3
