# Supplementary material for: Crosstalk between heterotrimeric G protein-coupled signaling pathways and WRKY transcription factors modulating plant responses to suboptimal micronutrient conditions
Source: J Exp Bot. 2020 Feb 28;71(10):3227–39. doi: 10.1093/jxb/eraa108 (PMC7260721; doi:10.1093/jxb/eraa108)
Supplement: eraa108_suppl_supplementary_figures_S1-S6_tables_S1_S4-S5 [file eraa108_suppl_supplementary_figures_s1-s6_tables_s1_s4-s5.pdf]

Supplementary Information

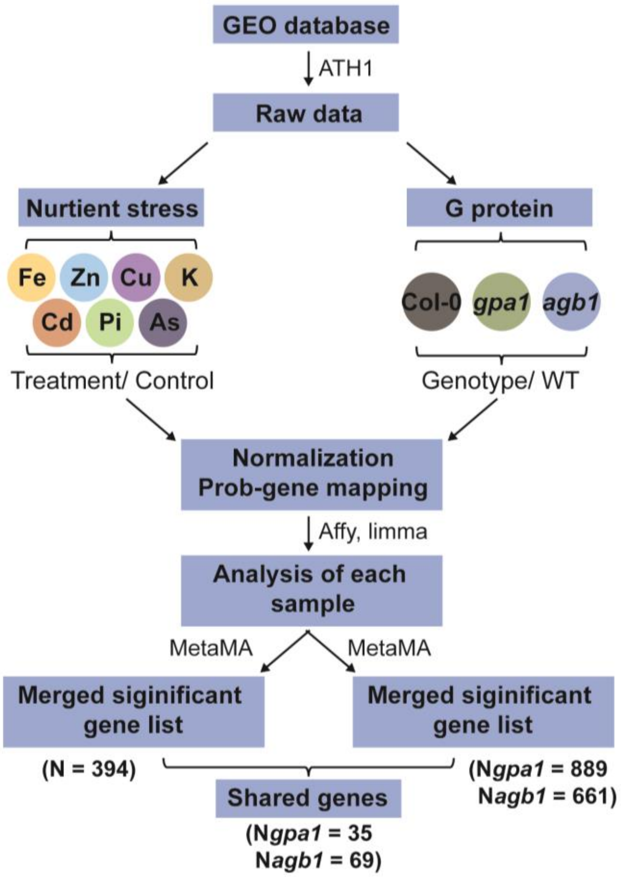

Figure S1 Bioinformatics pipeline for meta-transcriptome analysis. Transcriptome datasets were obtained from previous researches studying the effects of nutrient stresses or G protein mutations. The raw data were processed with R packages ‘affy’ and ‘gcrma’ for quantile normalization and background correction, followed by the identification of differentially expressed genes (DEGs) with ‘limma’ R package. ‘MetaMA’ R package was used to reduce the size effect and to get the moderate P-value. The numbers in parentheses represent DEGs identified from this informatics pipeline.

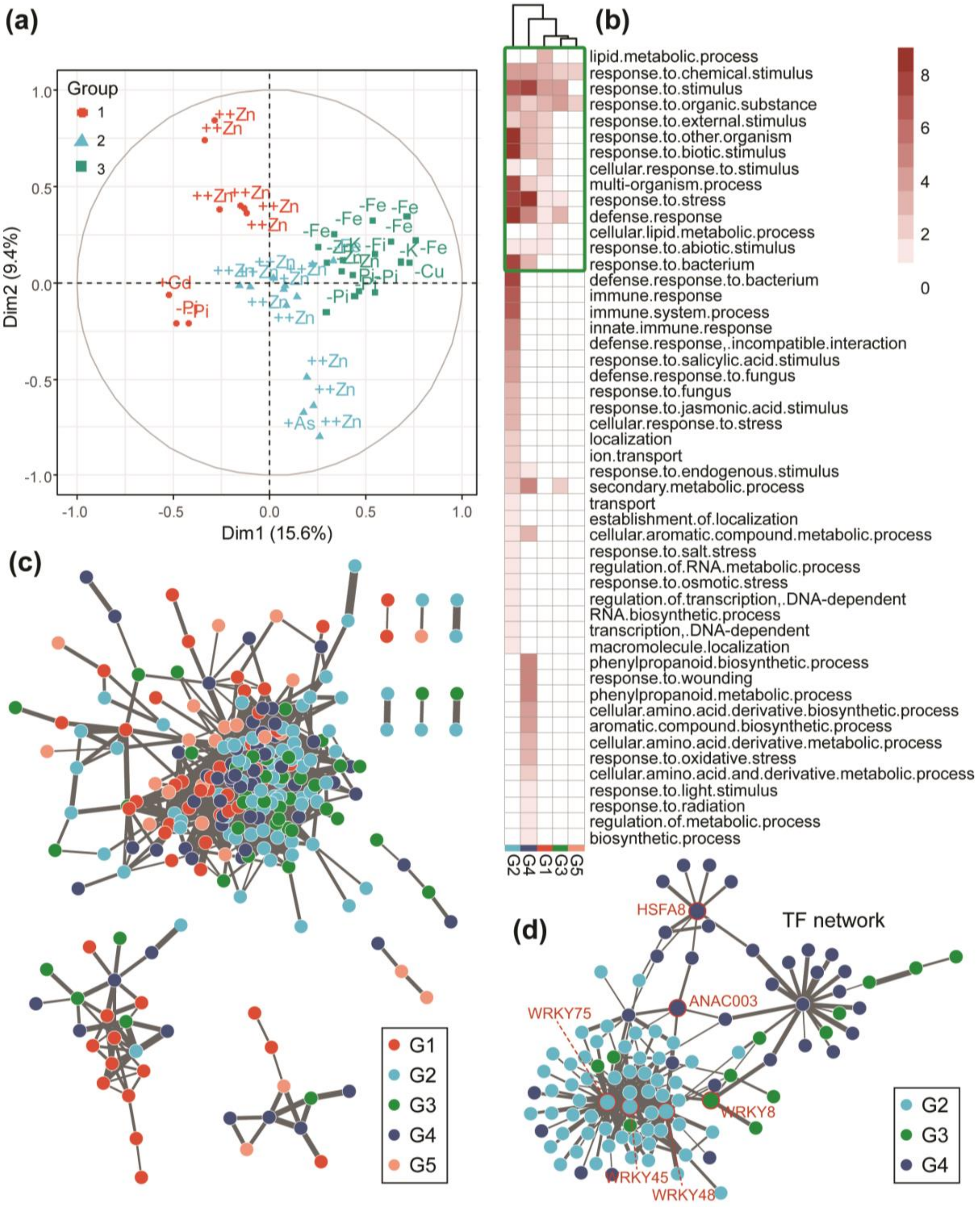

Figure S2 Transcriptional regulators for different types of nutrient stresses. (a) PCA analysis of common nutrient responsive genes from each dataset. Three groups were defined with a k-means clustering method. **(b)** GO analysis of the common nutrient responsive genes. GO terms of biological process are shown. Color bar represents $-\log_{10}$ (FDR) values. Fig 1b shows the same data with a more stringent threshold, $-\log_{10}$ (FDR)

greater than 1. **(c)** Co-expressed gene network of common nutrient responsive genes constructed by WGCNA. Edges represent possible correlation of gene expression patterns among paired genes with the adjusted p-value < 0.01 and the weight > 0.6. Node colors represent five groups defined in **(a)**. **(d)** Semi-supervised network of TF genes constructed by GENIES3 from the common responsive genes. The hub nodes (circles with red outline) represent the central TFs which regulate the co-expressed genes. Node colors represent five groups defined in **(a)**.

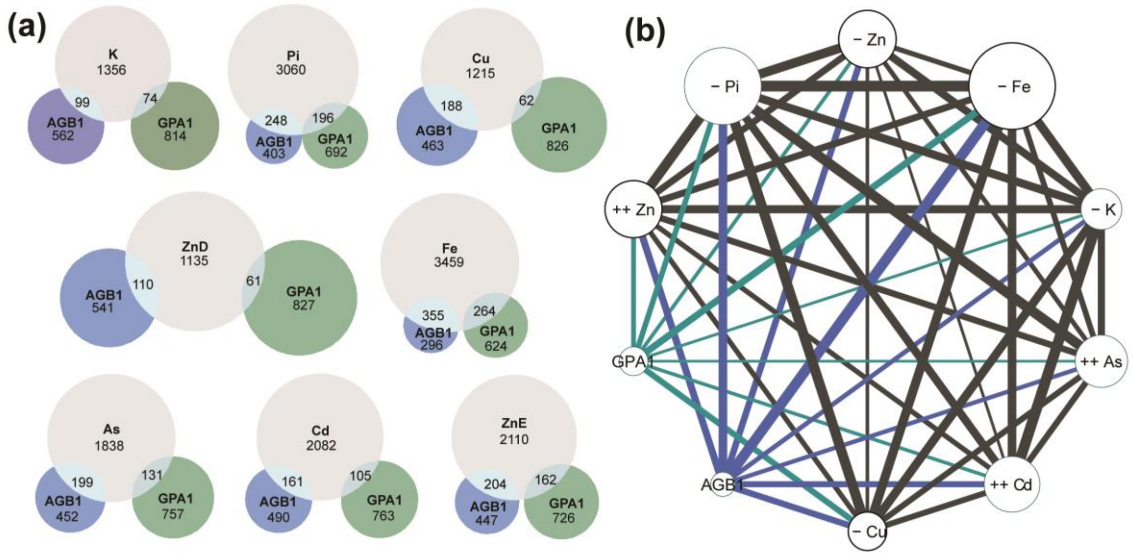

Figure S3 Overlapping degree between *gpa1*-specific and *agb1*-specific genes with each nutrient-specific genes. (a) Venn diagrams showing *gpa1*- and *agb1*-specific genes (green circles and blue circles) overlapped with individual nutrient-stress related genes (gray circles). All metal DEGs, except for the K^+ DEGs, and *gpa1* or *agb1* DEGs are significantly overlapped, as determined by hypergeometric test. For example, *agb1-2* vs zinc overlapping genes, p-value < 1 x 10^{-5}; *gpa1-3* vs zinc overlapping genes, p-value < 0.05. **(b)** Correlation network of *gpa1*- or *agb1*-specific genes with all analyzed nutrient-stress related genes. Edge width represents the degree of overlap and vertex size represents the number of differentially expressed genes identified in each condition. The edges connected to the vertexes, GPA1 or AGB1 (*gpa1*-specific or *agb1*-specific genes respectively), are represented in light green or blue.

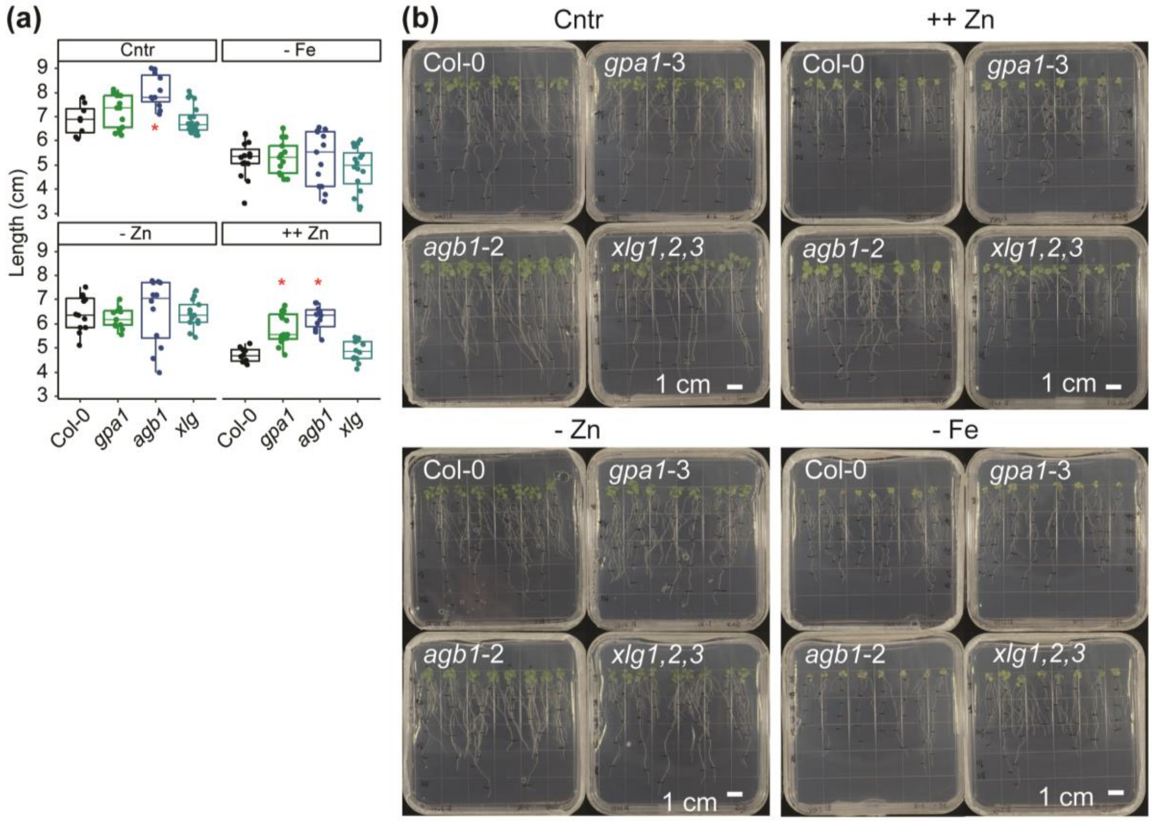

Figure S4 Root length of Col-0 and G protein mutants under iron, zinc deficiency and zinc excess in Arabidopsis. (a) Root length of Col-0, *gpa1-3*, *agb1-2* and *xlg1,2,3* mutants under control, Fe deprivation, Zn deprivation and Zn excess conditions. Raw data and a box plot are displayed for each genotype. One-way analysis of variance (ANOVA) followed by the Tukey's multiple comparison test was used to determine significant differences. N = 10-12 plants for each genotype under each treatment. *p < 0.05, **p < 0.01. (**b**) Images of Arabidopsis roots grown on vertically inclined plates with control or nutrient stress media. Note that representative root images are presented in Fig 3a.

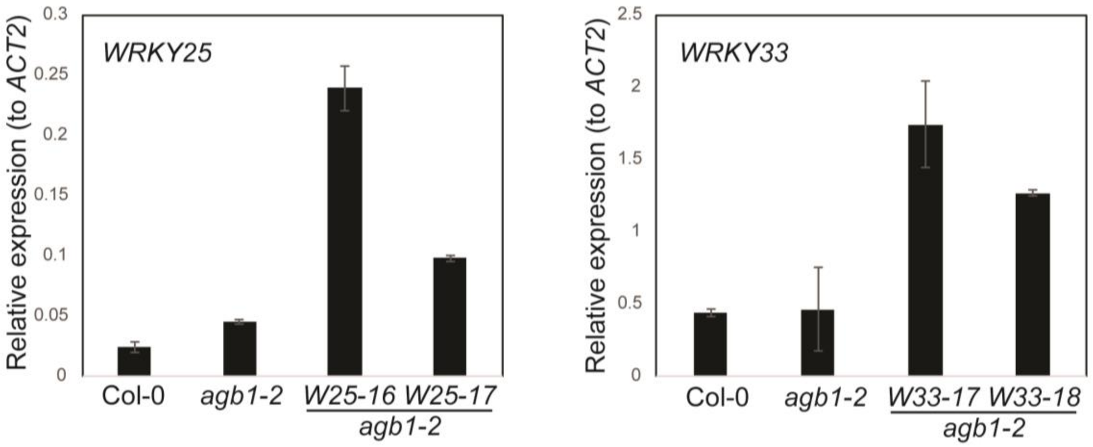

Figure S5 Expression of *WRKY25* and *WRKY33* of WRKYs OE lines in *agb1-2* background. Quantitative RT-PCR analyses of *WRKY25* and *WRKY33* genes in T1 generation of *WRKY* OE transgenic plants. Black and red dash lines indicated the expression level of *WRKY25* and *WRKY33* in Col-0 and *agb1-2* plants, respectively.

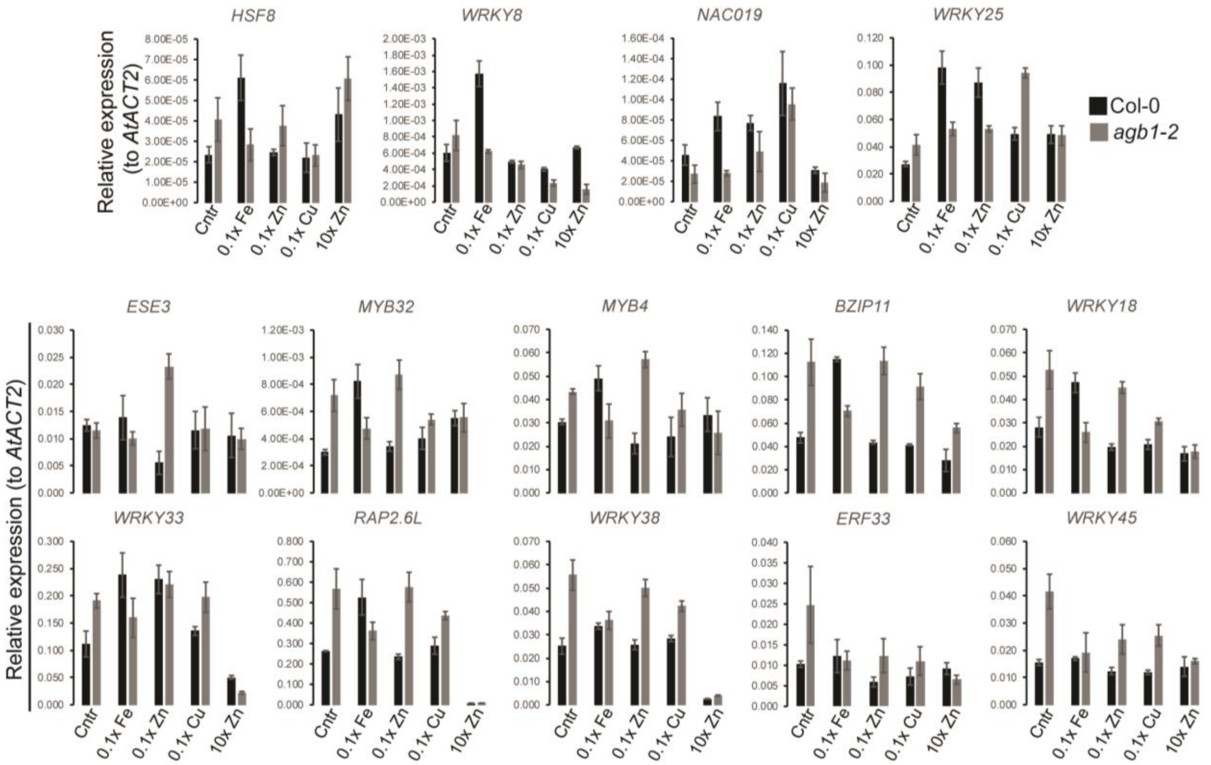

Figure S6 Quantitative RT-PCR analyses of TFs selected from commonly expressed gene list. Five day-old seedlings of Col-0 and *agb1-2* were grown under iron, zinc or copper deficient or zinc excess condition, respectively. Data show expression levels relative to a reference gene, *AtACT2*. The expression data are standardized and presented in the heatmap in Fig 1f and 1g. N = 3.

Table S1 Sources and experimental conditions of microarray data used for this study

Table S2 List of common nutrient responsive genes

Table S3 List of *gpa1*- and *agb1*-specific genes

Table S4 List of primers used for qRT-PCR experiments

Table S5 Shoot and root phenotypes under low or excess iron and zinc conditions.

Table S1. Sources and experimental conditions of microarray data used for this study. Refer to Materials and Methods for more information on transcriptional meta-analysis and selection of microarray datasets.

Condition	DEGs	Studies	GEO series	References
**Nutrient stress**				
-Pi	3504	4	GSE52046, GSE33790, GSE66925, GSE16722	(Bustos et al., 2010; Puga et al., 2014; Shukla et al., 2017; Woo et al., 2012)
-Fe	5853	6	GSE63946, GSE77806, GSE51692, GSE92716, GSE40076, GSE24348	(Kumar et al., 2017; Pan et al., 2015; Schuler et al., 2011; Sivitz et al., 2012; Zamioudis et al., 2015)
-K	1541	2	GSE6852, GSE6160	-
-Zn	2496	5	GSE96589, GSE77286, GSE31778, GSE5738, GSE5613	(Azevedo et al., 2016; Deinlein et al., 2012; Filatov et al., 2006, 2007)
+Zn	2452			
+Cd	2348	2	GSE94314, GSE22114	(Fischer et al., 2017; Li et al., 2010)
+As	2116	1	GSE49037	(Castrillo et al., 2013)
-Cu	1215	2	GSE91004, GSE42642	(Perea-García et al., 2013)
	Common: 394	Total: 22		
**Time course**				
-Pi	3869	1	GSE25171	(Lin et al., 2011)
-Fe	3465	2	GSE10576, GSE10502	(Dinneny et al., 2008)
		Total: 3		
**Genotype**				
agb1-2	661	4		

gpa1-3	889		GSE19520, GSE6171, GSE34667, E-MTAB-641	(Booker et al., 2012; Delgado-Cerezo et al., 2012; Pandey et al., 2010)
		Total: 4		

Table S4. List of primers used for qRT-PCR experiments

Gene		5'-3'
AtACT2	F	TGTGCCAATCT ACGAGGGTTT
	R	TTTCCCGCTCTGCTGTTGT
AtWRKY25	F	ACCTCTTCCGATTTCACA
	R	GTTCCATTAAAGCCTTGC
AtWRKY33	F	TACGAAGGGAAACACAACCA
	R	AAGGCCCGGTATTAGTGTTG
AtWRKY18	F	CGT GCC TAC TGA AAC ATC GGA C
	R	GTAAGCTCTAGGTGACGGGTTGTC
AtWRKY38	F	CGC CAT GCG GTT GAA GAG
	R	TAACTTGAAAGCGGTCCACCAT
AtWRKY8	F	ATGATCTCTTCCGTGTGCCA
	R	ATCATCAAGGCTCTTGTTTGAAGA
AtWRKY45	F	TGCACAGAAGAAGGATGCAG
	R	TGGTATGTCGTCACCACCAC
AtPHT1;1	F	CCTTTGGGTTCCTATATGCG
	R	TAACCTCAGCCTCACCAGAG
AtIRT1	F	TTAGGTCCCATGAACG
	R	AAGCTTTGATTCACGGTT
AtRAP2.6L	F	CAAGGCCCTACTACCACCACAA
	R	GGTCGAGGAGGAGGTGAGTTC
AtHSFA8	F	CCCAAATTCTCTTCATTTTTCC
	R	AAACTTTAGATGAAATTTTGAATTCTG
AtNAC019	F	TCAAGACCTAACCGGGTTGCCGGATC
	R	TCTCTAGCATTCGCGATTCCATTATCG
AtBZIP11	F	TCTCATGGCGTCTTCTGATG
	R	AACGGCCCCATTTCTTTG
AtERF33	F	ATCATGGGCGATGCGAATAA
	R	GCGAGAAATATTCGGTCTGGTT
AtNAS1	F	AAC GAC GTC ATA TTG GTC AAG
	R	ATC TTC CAC ACA ACG GAC G
AtMYB4	F	TTTCGAAGCTTGGAGATGAAA
	R	ACTTTCAAATTATCTAGGCAAAAGTT
AtMYB32	F	CTTTGGAAATGAAATGAAATGAAA
	R	TGAGAACCATGAACGAGTCTG
AtESE3	F	GCCATTCCTACTTCCTCTTC
	R	TCTCCGTTACTCCTCTGTTC
AtCOPT2	F	CCTTTCGTATTTGGTGATGCT
	R	AAACACCTGCGTTAAAGGAC
AtYSL2	F	TCTTATAAATGGATTTCATACTA
	R	AATGCCCAAAAGAAACTCAAA
AtZIP2	F	CGCTTGGAGAAACCTATGGA
	R	CGACACCTATGGGACTCGAT
AtFRO2	F	GCGACTTGTAGTGCGGCTATG
	R	CGTTGCACGAGCGATTCTTG
AtZIP3	F	CTCCTTCTCATCGCCGTCGT
	R	CGAGCTCCGGCTTTGTTTTC

AtZIP4	F	GGCTGCATCTCTCAGGCACA
	R	GGCCACTGCAGTTCCAATCC
AtIRT3	F	ACACGGACATGGACACGTACAC
	R	CAATTCCAAGCTCCAGAACCTGAG
AtBZIP19	F	CGTGCTTCCATGTCCACACC
	R	CCCGGTTTCCCAAAGGTCTC
AtFIT	F	ACCTCTTCGACGAATTGCCTGACT
	R	TTCATCTTCTTCACCACCGGCTCT
AtBZIP23	F	TAATCAGCTGTTGAAGAGGT
	R	TCATGTATGAGTAAGGCACG
AtPAP24	F	ACACGATTGGAGAGAAGGCA
	R	AACCAAGGACACGATGAGCT
AtSEN1	F	AGGAAATGTTGCAGCAGAGG
	R	CGTTGATGGCTCTAGTCGGA
AtASN1	F	GGAATATTTGGGGACGGTGC
	R	CGGGACATCAAGAACATCGG
AtHKT1	F	TCTTGGAGTGACGGTGCTAG
	R	CAGAGGTCCATTCAAAGGCG

Table S5 Shoot and root phenotypes under low or excess iron and zinc conditions. Data shown are the mean value ± SD with significance symbols determined by two-way ANOVA follow by Tukey's multiple comparison test. * or ** indicates significant difference from the Col-0 data with the P value less than 0.05 or 0.01, respectively. Experiments for shoot phenotypes were repeated 3 times with more than 50 plants per experiment. Root length data is from Figure S4.

	Growth arrest (%)	Black meristem (%)	Leaf area (mm^2)	Chlorophyll index	Root length (cm)
			**Control**		
**Col-0**	2.04 ± 0.42	4.08 ± 0.15	7.32 ± 1.12	136.50 ± 8.51	6.89 ± 0.46
gpa1-3	1.21 ± 0.32	2.08 ± 0.27	7.43 ± 0.32	155.97 ± 10.17	7.27 ± 0.74
agb1-2	2.38 ± 0.35	7.14 ± 0.21*	7.99 ± 1.51	157.71 ± 9.58	8.07 ± 0.84*
			**- Zn**		
**Col-0**	12.73 ± 1.28	23.64 ± 0.87	5.71 ± 0.40	139.57 ± 9.45	6.44 ± 0.32
gpa1-3	2.22 ± 1.17**	15.56 ± 1.04*	6.37 ± 0.35	147.76 ± 7.21	6.23 ± 0.21
agb1-2	22.50 ± 1.16**	67.50 ± 1.24**	4.14 ± 0.33*	138.72 ± 5.12	6.50 ± 0.28
			**++ Zn**		
**Col-0**	31.25 ± 1.31	14.58 ± 0.91	6.84 ± 0.75	108.27 ± 5.24	4.71 ± 0.78
gpa1-3	11.11 ± 1.27*	6.67 ± 1.18*	6.52 ± 0.51	133.28 ± 4.86*	5.81 ± 0.35*
agb1-2	15.00 ± 1.18*	47.50 ± 1.21**	5.56 ± 0.31*	126.65 ± 3.47	6.23 ± 0.72*
			**- Fe**		
**Col-0**	34.62 ± 2.45	5.77 ± 0.48	5.07 ± 1.08	81.56 ± 5.12	5.25 ± 0.31
gpa1-3	14.58 ± 3.78*	2.08 ± 0.94*	5.51 ± 1.01	120.46 ± 5.76*	5.31 ± 0.27
agb1-2	9.30 ± 3.94*	9.30 ± 0.81*	6.12 ± 1.20	120.10 ± 4.18*	5.28 ± 0.21
